# Integration and scale-up of a primary healthcare-based chronic wound care package for persons affected by skin-neglected tropical diseases and other conditions in Ethiopia: a protocol for an implementation research study

**DOI:** 10.1186/s12875-026-03259-9

**Published:** 2026-03-09

**Authors:** Mersha Kinfe, Maya Semrau, Asrat Mengiste, Oumer Ali, Tigest Ajeme, Stephen Bremner, Natalia Hounsome, Vasso Anagnostopoulou, Malcolm Brewster, Lawrence Rugema, Eiman Siddig Ahmed, Agumasie Semahegn, Abebaw Fekadu, Gail Davey

**Affiliations:** 1 Center for Innovative Drug Development and Therapeutic Trials for Africa (CDT-Africa), Addis Ababa University, Addis Ababa, Ethiopia; 2 Centre for Equitable Global Health Research, Department of Global Health and Infection, Brighton and Sussex Medical School, Brighton, BN1 9PX UK; 3 Armauer Hansen Research Institute, Addis Ababa, Ethiopia; 4 Department of Primary Care and Public Health, Brighton and Sussex Medical School, Brighton, BN1 9PX UK; 5 School of Public Health, College of Health Sciences, Addis Ababa University, Addis Ababa, Ethiopia; 6 Ferry Road Health Centre, Rye, East Sussex, UK; 7 School of Public Health, College of Medicine and Health Sciences, University of Rwanda, Kigali, Rwanda; 8 College of Health and Medical Sciences, Haramaya University, Harar, Ethiopia

**Keywords:** Chronic wounds, Primary healthcare, Neglected Tropical Diseases, Low-income countries, Ethiopia

## Abstract

**Background:**

The profound impact of wounds on the quality of life of those affected is often underestimated. Chronic wounds impose substantial burdens on individuals and communities in terms of disability, mental distress, stigma, and economic productivity losses. To effectively address these challenges, an integrated and comprehensive approach to primary healthcare-based chronic wound care prevention and management is essential. This implementation research study aims to assess the integration and scale-up of a comprehensive package of primary healthcare-based wound care and psychosocial support for persons affected by chronic wounds caused by NTDs and other conditions in selected districts in Ethiopia.

**Methods:**

The study will be implemented in Central Ethiopia and Amhara Regions in three stages, utilizing a mixed-methods approach to co-develop a comprehensive care package and progressively implement the care package building on learnings from successive stages of implementation. Stage 1 will encompass the co-development of a holistic wound care package and strategies for its integration into routine primary health services. Stage 2 will involve a pilot study in one sub-district, to establish the care package’s adoption, feasibility, acceptability, fidelity, potential effectiveness, readiness for scale-up, and costs. Stage 3 will involve the scale-up of the wound care package and its evaluation in several districts.

**Discussion:**

This implementation research on integration and scale up of chronic wound care within primary health services will substantially improve access to care and support for affected persons in Ethiopia. It will also have critical contribution to the national wound care and integrated early detection, management and reporting of chronic wound due to skin-NTDs and other chronic health conditions government initiative in the primary health care system of Ethiopia. The finding from this embedded implementation research will be disseminated to relevant stakeholders using a variety of channels to inform policymaking and program design. Persons affected by chronic wounds and their family, healthcare providers, and program planners will benefit from this study.

## Background

Many Neglected Tropical Diseases (NTDs) can cause wounds and other skin complications. Wounds are among the most frequently encountered skin problems in rural settings in low- and middle-income countries (LMICs), where skin NTDs are also endemic [1, 2]. Wounds are among the major causes of visits to hospitals in Africa, accounting for 30–42% of hospital attendance and 9% of deaths every year [3]. However, they are also among the most underreported health challenges in many parts of Africa, probably partly because of limited access to health care services. Over 60% of the population of the African continent resides in rural areas, with up to 80% facing geographical barriers to accessing modern healthcare [4].

The causes for wounds are numerous, varying markedly by age group, environment, and occupation. They range from trauma, burns and bacterial infections to non-communicable diseases (NCDs) such as peripheral arterial disease and diabetes [1, 2]. Fifteen to twenty five% of patients with diabetes develop a diabetic foot ulcer in their lifetime [5, 6] and up to one-third of patients in acute and long-term care develop a pressure ulcer [7]. In most parts of Africa, wounds are caused by NTDs, assaults, road crashes, occupation-related injuries (e.g., at construction sites, on farms, and domestic accidents), animal bites, burns, surgery, and diseases resulting in acute or chronic soft tissue damage. NTDs that can cause wounds and other skin complications in Ethiopia include leprosy, cutaneous leishmaniasis, lymphatic filariasis (LF), podoconiosis, onchocerciasis, snakebite, and guinea worm, as well as ectoparasites such as scabies and tungiasis.

Studies investigating wounds in resource-limited settings are scarce. While chronic wounds impose huge burdens on affected individuals and communities in terms of disability [8], mental distress [9], depression [10], stigma [11–14], and loss of economic productivity [8, 15–17], the impact that wounds can have on the quality of life of those affected is under-recognized [18–22]. Furthermore, findings from cost analyses carried out in high-income countries (HICs) show a strikingly high cost burden [23, 24], and this also needs to be assessed in resource-limited settings.

High-quality and timely wound management is vital to prevent infections and disabilities. Unfortunately, wound management is often expensive and ineffective in the areas where NTDs occur. In these settings, the number of health professionals is limited, health workers are minimally trained in providing curative care, and self-care is not sufficiently implemented. Skills in evaluating abnormal signs and symptoms, such as when to stop using topical antiseptics, when to suspect secondary infection and when to suspect malignant alteration are lacking. There is limited training and the need for expertise in wound care is not well recognized. A study conducted in Ghana and Benin interviewing healthcare personnel dealing with the wound management of Buruli ulcer patients reported that standards of wound care differed greatly both between personnel and between institutions [25]. Variations in wound care may derive from a lack of formal wound care education, limited high-quality evidence, inconsistent guidelines, and the diversity of clinical techniques used to support wound healing.

Multiple gaps in wound care have also been identified in Ethiopia, including limited physician knowledge and poor use of best practices [26]. A survey conducted among nurses in Ethiopia found their knowledge and practice of wound care to be poor. The study found important gaps in knowledge and alarming lack of evidence-based practice. In addition, unfavourable attitudes among nurses towards wound care were identified. Updating nurses’ knowledge and creating favourable attitudes through training on prevention and management of foot care was recommended [6]. This might be achieved through provision of structured pre-service and in-service training and education programs on wound care. Integrating chronic wound care training into pre-service education for nurses and health officers requires a shift from traditional theoretical lectures to structured, competency-based curricula that bridge the “theory-practice” gap. Existing gaps in knowledge and skills of mid-level health workers need to be addressed to ensure that the proposed training is relevant and aligns with local priorities, and that the education is directly linked to health service needs and clinical practice. Engagement with relevant Ministries is also important. In addition, establishing and putting in place wound healing and care guidelines and assessment tools in health facilities and conducting further qualitative research on the levels of wound care practices is recommended [26].

Integrated wound management focuses on sustainable prevention and care of wounds in settings with limited resources. It comprises identification, treatment and preventive measures. Wound care is a cross-cutting component in the management of a number of skin-NTDs (such as leprosy, Buruli ulcer, yaws, cutaneous leishmaniasis, tropical ulcers, LF and podoconiosis) and can be delivered through the same individuals in the same health care settings. In many co-endemic areas, leprosy and Buruli ulcers are managed in the same facilities [27]. In Ethiopia, an integrated approach for LF and podoconiosis morbidity management has been demonstrated to be feasible [28].

Consultation with affected persons and implementer groups in Ethiopia suggests that development and evaluation of an integrated wound management service applicable across skin conditions would be enormously beneficial not just for persons affected by podoconiosis, LF and leprosy, but also for those with cutaneous leishmaniasis, diabetes and injuries. This is in line with the cross-disease emphasis of the new World Health Organization (WHO) Roadmap for NTDs 2021–2030 [29].

Innovative ways of ensuring support, including use of mHealth (mobile health) tools, tele-dermatology, and online training courses, may also be relevant. These innovative technological methods can help to bridge the gap between the burden of skin diseases (including chronic wounds) and the lack of capable staff in resource-poor settings by bringing essential health services from central settings closer to peripheral levels [30]. Opportunities for low-cost tele-dermatology applications will continue to increase, making it feasible for any provider in any setting to be connected to a tertiary hospital dermatologist, surgeon or other appropriate specialist. Incorporating telehealth into wound care delivery not only facilitates more timely patient access, but can be a more efficient utilization of wound specialists [31]. Recent literature indicates that telehealth facilitates earlier assessment and treatment of wounds and improves patient outcomes. Telehealth may be especially beneficial to people who reside in rural areas [32, 33] because it may reduce their need to travel long distances (and thus decrease travel-related costs) to specialty wound care clinics [34]. The use of telehealth for wound care may also reduce the overall number of clinic visits that patients require, which, in turn, may reduce related costs to the health care system [35] .

A comprehensive review of published papers in the field of chronic wound care revealed a low proportion of global publications with high quality, robust information [36]. Our observations identify a number of issues relating to the management of chronic wounds in Ethiopia including:

- The continued use of outdated practices which do not promote healing;
- Limited access for patients to effective treatment that would heal their wound;
- Lack of a coordinated approach to chronic wound management.

Shortcomings of existing services include a lack of evidence-based practice, insufficient resources, and in particular, poor understanding of the true cost to society and service providers.

In Ethiopia, high priority needs to be given to persons affected by chronic wounds. This is therefore an opportune time to address the challenge of integrating a comprehensive package of chronic wound care interventions into routine primary health services. The Health Extension Programme, rolled out successfully in Ethiopia since 2006, now boasts more than 38,000 community-based Health Extension Workers (HEWs) and a supervisory system to support them. Their reach has been extended through the women-centred Health Development Army (HDA), members of which link ‘model families’ with five other households to implement health initiatives [37]. These cadres are ideally placed to offer simple, low-tech wound care and psychosocial support to people living with diseases associated with chronic wounds in Ethiopia [38], and to refer patients who need more specialised wound care. The Ethiopian Federal Ministry of Health (FMoH) is aware of the nature and scale of the problem and has requested implementation research to guide two ‘axes’ of integration: i) development of an integrated package of physical and psychosocial care interventions for persons with chronic wounds, and ii) subsequent integration of this comprehensive wound care package into government-run primary health services.

To address this, the ‘Excellence in Disability Prevention Integrated across NTDs’ Part II (EnDPoINT II) implementation research study is running in Ethiopia from 2021 to 2026, funded by the National Institute for Health and Care Research (NIHR) in the UK. The aim of this implementation research study is to integrate and scale up a comprehensive package of primary health care-based physical and psychosocial care for persons affected by chronic wounds due to NTDs and other conditions in selected districts in Ethiopia across three project stages.

## Methods

### Conceptual framework for study

This study is an implementation research study in that: its research questions have a strong focus on implementation strategies; it includes a wide range of implementation outcomes; and it is being conducted in real-world settings and populations [39]. The conceptual framework used for the study will be the Medical Research Council (MRC) framework for complex interventions [40], embedded within a ‘Theory of Change’ approach. The MRC framework for complex interventions has been used widely and proposes four phases in the development and evaluation of interventions:

i. development,
ii. feasibility/piloting,
iii. evaluation, and
iv. implementation.

These phases are considered to be an iterative rather than a linear process. ‘Theory of Change’ (ToC) has been defined as a “theory of how and why an initiative works” [41]; it enhances the MRC framework for developing and evaluating complex interventions through its theory-driven approach, which can be incorporated into and provide practical guidance for the different phases of the MRC framework [41]. Within this study, the ‘ToC’ approach will only be used within two of the four phases of the MRC framework: the development and piloting phases, since for these two phases, there is good evidence for the practical feasibility and usefulness of an embedded ‘ToC’ approach [42], whereas this is not yet the case for the phases on implementation and evaluation.

### Study design

The three project stages are as follows:

*Stage 1* corresponds to Phase 1 of the MRC framework on complex interventions – the development of the comprehensive wound care package. Various activities will be carried out to inform the development of the care package and strategies for its integration into the routine primary health care delivery system: (1) A scoping review of peer-reviewed and grey literature will be conducted to gain an understanding of documents relevant to the development of the comprehensive wound care package; (2) A situational analysis and resource assessment will be conducted to provide the local context for the development and implementation of the care package in the selected districts in Ethiopia, and to identify the resources available for the implementation of the care package; (3) Three ‘ToC’ workshops will be held with members of the EnDPoINT II research team and key stakeholders, to identify the desired outcomes, indicators, interventions, and measurement of the outcomes for the wound care package, and to encourage stakeholder buy-in to the study; (4) Key informant interviews and focus groups will be carried out with key stakeholders, to assess the care package’s feasibility (i.e., the extent to which the intervention can be carried out within the routine health system), acceptability (i.e., the perception among stakeholders that the comprehensive wound care package is agreeable), and appropriateness (i.e. the perceived fit or relevance of the care package to key stakeholders). Informed by the above steps, the comprehensive wound care package consisting of physical and psychosocial care interventions at the healthcare administration, health facility and community level will be developed.

*Stage 2* corresponds to Phase 2 of the MRC framework on complex interventions – piloting of the comprehensive wound care package in one selected sub-district in Ethiopia. This will enable study of the factors that influence integration of the care package and assessment of the wound care package’s adoption, feasibility, effectiveness, fidelity, and coverage within the government-run health system and the roles of key health system actors. Mixed methods will be employed for this, including pre-post collection of quantitative survey data (affected persons and community members); pre-post knowledge, attitudes and practice (KAP) questionnaires and an evaluation of training (health staff); as well as qualitative in-depth interviews (affected persons and their caregivers).

*Stage 3* corresponds to Phases 3 and 4 of the MRC framework and will involve scale-up and evaluation of the wound care package in several districts in Ethiopia based on learnings from the previous two stages.

### Setting

The EnDPoINT II study is planned to be implemented in two zones: East Gurage in Central Ethiopia and Awi in Amhara Region in North-Western Ethiopia. These districts were selected based on the co-endemicity of diseases causing lymphoedema and after consulting with the Ministry of Health.

Based on the last census conducted by the Central Statistical Agency of Ethiopia (CSA) in 2007, Gurage has a total population of 1,280,483 [43]. The six largest ethnic groups reported in Gurage Zone were the Gurage people (82%), the Mareqo or Libido (4.3%), the Amhara (3.4%), the Kebena (3.3%), the Silt’e people (2.7%), and the Oromo (1.7%); all other ethnic groups made up 2.6% of the population. Gurage was spoken as a first language by 80.5% of the population, 5.3% spoke Amharic, 4.1% spoke Libido, 3.2% spoke Kebena, 3% spoke Silt’e, and 1.1% spoke Oromo; the remaining 2.8% spoke other primary languages. The majority of inhabitants (51%) were reported to be Muslim, while 41.9% practised Ethiopian Orthodox Christianity, 5.8% were Protestant, and 1.3% Catholic. 79% (79%) of all eligible children were enrolled in primary school, and 12% in secondary schools [43].

Based on the 2007 CSA, Awi Zone had a total population of 982,942. The two largest ethnic groups reported were the Awi (59.8%), a subgroup of the Agaw, and the Amhara (38.5%); all other ethnic groups made up 1.7% of the population. Amharic was spoken as a first language by 53.4% of the population, and 45% spoke Agew-Awigna; the remaining 1.6% spoke other primary languages. 94.4% practiced Ethiopian Orthodox Christianity, and 4.5% of the population were Muslim. 72% (72%) of all eligible children were enrolled in primary school, and 16% in secondary schools [43].

### Participants

For Stage 1, as no primary research will be conducted for the scoping review and situational analysis, no participants will be recruited for these aspects (though some of the information for the situational analysis/resource assessment may be obtained by contacting key officials, if necessary, for example by phone). For the ‘ToC’ workshops, members of the EnDPoINT II research team in Ethiopia participated in three workshops held to date. Participants of the second ‘ToC’ workshop included members of the wider EnDPoINT II Consortium, and other key stakeholders such as current NCD and NTD experts (including Non-Governmental Organization (NGO) implementers), policy-makers, health service planners, health workers, community leaders, and members of affected persons’ organisations. Participants in the key informant interviews and focus groups during Stage 1 will include key stakeholders who already are, or who will be involved as part of the implementation of the study, in the provision and/or receipt of NCD/NTD care, for example dermatologists, surgeons, HEWs, NGOs working within NCDs and NTDs, primary health care workers, health managers and decision-makers, policy-makers, community leaders, traditional and religious leaders, and affected persons and their families.

During Stages 2 and 3, the pilot study and scale-up of the wound care package, a wide range of stakeholders at each of the three levels of the health system (health care organization, health facilities, and the community) will receive training to implement the various components of the care package. This will include staff at the health care organization level, zonal and district level health office staff, psychiatric nurses, senior health care workers, senior pharmacists, pharmacy staff, health centre staff (health officers and nurses), HEWs, and members of the community (see ‘Procedure’ section below for further details). They will also be invited to participate in pre-post KAP) questionnaires, and an evaluation of the delivery of the training.

In addition, affected members of the community, i.e. people with chronic wounds, as well as their families and their communities will be included in the study as recipients of the care package interventions. Affected people may also be involved in some of the delivery of the training packages or interventions as experts by experience to provide a ‘lived testimony’ of living with chronic wounds and include a social contact element in the training and interventions. Furthermore, both affected persons (patients) and community members will take part in a quantitative pre-post survey, which will be administered to each of these two groups before and after implementation of the care package (with several follow-up points). To gain deeper insight into the lived experiences of persons affected by chronic wounds and their caregivers, in-depth qualitative interviews will also be conducted with them alongside the quantitative data collection.

Adults aged 18 or over will be included in the study. All aspects of the study will be conducted in either Amharic or English, depending on which language is most appropriate for each participant group. When necessary, all study materials, including participant information sheets and consent forms, will be translated into Amharic, the official language of Ethiopia, which is spoken and understood by the majority of people in the country, including those in the study areas.

### Sampling and sample size

Key stakeholders will be identified and recruited using purposive sampling techniques for the ‘ToC’ workshops in Stage 1, the key informant interviews and focus groups in Stages 1 and 2, and the training elements in Stage 2. Participants will be selected based on their role and position. The EnDPoINT II research team will initially contact key stakeholders to invite them to participate. Snowballing techniques may also be used, where each identified key stakeholder will be asked to recommend other potential participants. The number of participants taking part in the ‘ToC’ workshops in Stage 1, and the key informant interviews and focus groups during Stages 1 and 2 of the study, will be guided by the number of key stakeholders that are identified. A sample size calculation is not appropriate for these qualitative data collection methods; rather we will follow a data saturation approach, i.e. data collection will continue until sufficient information has been obtained or where further interviews fail to generate new themes. However, the number of participants during the ‘ToC’ workshops and focus groups will not exceed *N* = 12, to ensure that all participants have the opportunity to speak. For the training elements in Stage 2 of the study, the number of participants will be established based on the number of people who are suitable and available for training.

For the before-and-after (pre-post) collection of quantitative data through the patient and community surveys in Stage 2 of the study (with data collection points at baseline before implementation of the care package, and at 3- and 12-months follow-up), paired data from 120 patients will be collected in the selected sub-districts; 60 from each district [44]. For the community survey, 200 participants will be included. These numbers will provide good precision of estimated retention proportions that will be used to inform the sample size and other design elements in Stage 3 of the study [44].

### Inclusion/exclusion criteria

Participation in all aspects of the study will be voluntary. Participants in the second ‘ToC’ workshop, the key informant interviews and focus groups, those who are taking part in the training evaluations, and participants of the patient and community surveys, will be required to give their informed consent to take part (at baseline). If a person does not consent to take part in any of these research evaluation activities, that person will be excluded. Participants will all be over 18 years of age. Participants must have adequate hearing to ensure effective communication. Individuals with severe hearing impairments that significantly hinder communication will be excluded. Participants must be in a stable health condition, meaning they are not acutely ill or experiencing severe pain that could interfere with their ability to participate fully in the study. Individuals who are currently experiencing acute illness or severe pain will be excluded.

### Care package description

The interventions included in the care package will be organised according to the three levels of the health care system in Ethiopia: the health care organization level which includes the Federal Ministry of Health, Regional Health Bureau, zonal health department and district health office; the primary health care facility level which includes primary hospitals, health centres and health posts; and the community level. The draft care plan may include the interventions shown in Table 1.

**Table 1:** Potential wound care package interventions

Domains	Descriptions
Clinical photography in chronic wound care using smartphones	There is a high level of smartphone ownership and usage among nurses and health officers working at health centres and primary hospitals in Ethiopia. These health workers will take clinical photographs after obtaining written consent from the patient mainly to gain advice from peers/consultants and for treatment and disease monitoring purposes. They will photograph the wound on initial assessment and repeat every four weeks or more frequently if the wound condition changes rapidly. The consulting hospital wound care practitioner will ask the healthcare nurse practitioner a series of basic wound assessment questions. A treatment plan will be developed based on visual examination of the wound and responses to the wound assessment questions.
Health care organization level	• High-level awareness-raising and mobilization: participatory workshop for zonal and district health bureau officials and community advisory board; • Programme management, i.e. working in partnership with key health care organization staff to implement the necessary structures and budget for the delivery of the care package; • Capacity-building, i.e. ‘Training of Trainers’ (ToT) for health care organization staff about wound care provision and supervision. Resources from the WHO (46) and other sources (38) provide rich information about managing wounds; • Formation of Public Advisory Group (PAG) with members of the community, NTD and NCD task force.
Primary health care facility level	• Awareness-raising amongst health facility staff and attendees: (i) workshop for facility-based staff by NTD/NCD focal person at health care organization level; (ii) posters in health centres; (iii) health education sessions by health facility staff for attendees of health facilities; • Case detection, assessment, and treatment and wound care initiation by health officers and nurses, including supply package, psychosocial support, and health education for patient self-care; • Capacity-building for facility-based health centre staff; • Supervision and mentoring for health centre staff; • Use of telehealth digital technologies, mobile health apps, and remote patient monitoring to support long-distance consultation on clinical health care. This study will clearly indicate the utilization of telehealth in the final care package. Digital pictures of wounds will be captured and used to assess improvements during follow-up patient visits. • Institution-based rehabilitation, e.g. reconstructive surgery, links with multi-disciplinary team, internist, surgeons and mental health care providers; • Supply chain management.
Community level	o Community awareness-raising: awareness-raising workshops for members of the community by HEWs and NGOs; o Information dissemination in the community by HEWs and NGOs (information leaflets and posters in health posts; posters/loudspeaker in the marketplace) on the common causes of chronic wounds; o Active case detection by HEWs; o Continuing care/follow-up home visits to patients and their families by HEWs; o Community-based rehabilitation, including home visits, workshops, patient associations and self-help groups; o Capacity-building for HEWs; o Supportive supervision for HEWs. Health Extension Workers will carry out the proposed chronic wound activities by integrating them into the existing programs rather than treating them as a stand-alone activity.

## Procedures

### Stage 1

#### Scoping review

To support the development of the wound care package and strategies for its integration into the routine health care delivery system in Ethiopia, a scoping review will be conducted on the implementation of primary health care and community-based physical and psychosocial interventions for the management of chronic wounds as well as lessons learnt from program implementation in low- and middle-income countries. This will include barriers and challenges to telemedicine/health for chronic wound care in LMICs. The scoping review will incorporate both publications listed in bibliographic databases as well as grey literature, including:

- Existing wound care training packages and materials from both LMICs and HICs;
- Documents relevant to the primary care provision of physical or psychosocial care for wounds, such as relevant project reports, programme documents and relevant publications in scientific journals;
- Any other relevant materials.

#### Situational analysis and resource assessment

To better understand the context of implementation and resources that may be available to support the implementation of the care package interventions, standard situational assessment and resource mapping tools will be used. The tools will be developed based on expert input from members of the EnDPoINT II Consortium and from previous situational analysis and mapping tools that have been used in other projects such as EnDPoINT I [45].

#### ‘Theory of change’ workshops

The main aim of the ‘ToC’ workshops is to build consensus and define the desired outcomes, indicators, interventions, assumptions and measurements for the comprehensive chronic wound care package, and to encourage stakeholder buy-in to the study [41, 46]. A ‘ToC’ map will be developed through a series of three workshops with key stakeholders, which will include policy makers, implementers, care providers, service users, community leaders and organizations.

The ‘ToC’ map developed during the workshops may need to be adapted further following the subsequent key informant interviews and focus groups, to account for the information provided on the wound care package’s feasibility, acceptability and appropriateness. Similarly, the ‘ToC’ developed during Stage 1 may need to be adapted following pilot-testing of the care package during Stage 2.

#### Key informant interviews and focus groups

The main aim of the key informant interviews and focus groups conducted in Stage 1 of the study will be to assess the feasibility, acceptability and appropriateness of the draft wound care package, including the key assumptions identified during the ‘ToC’ workshops [41].

Before we start stage two, we will evaluate the success criteria based on the assessment criteria of stage one as indicated in Table 2 below.

**Table 2:** Stage 1 success criteria and measures of assessment

Success criteria	Assessment of criteria
Wound care package that includes physical and psychosocial care for people with chronic wounds finalised for piloting, including guidelines and training materials	Wound care package including guidelines and training materials developed and agreed by the project team (based on the activities listed in the box below), and peer-reviewed and ‘approved’ by at least two wound care experts as well as one mental health expert (who can be internal to project)
Key elements identified that constitute optimal physical and psychosocial care for patients with chronic wounds	Following activities completed (to identify key elements for optimal wound care, learn lessons about integration of wound care, and develop strategies to facilitate its integration), and fed into the development of the wound care package: • Scoping review on wound care • Systematic review on telehealth • Situational analysis / resource assessment in study district(s) • Community Advisory Board meeting (at least one meeting in each study district) • Three ‘Theory of Change’ workshops involving a wide range of stakeholders • Qualitative work (focus group discussions and key informant interviews) with a wide range of key stakeholders
Lessons learnt about integration of wound care to date	
Strategies developed to facilitate integration of the care package into the routine health care delivery system	
Care package considered feasible, acceptable and appropriate by key stakeholders	• Qualitative assessment through focus group discussions and key informant interviews • (Internal) peer-review of the wound care package (see above) Of note: should the care package not be considered feasible, acceptable and appropriate by several key stakeholders (including Health Extension Workers), it will be revised until considered to be so.

### Stage 2

#### Pre-piloting in a health facility

As indicated in Table 2 above, after evaluation the success criteria for stage one an initial pre-pilot will be carried out during Stage 2, to assess the main training elements of the primary health facility and community components of the draft wound care training package, and evaluate their feasibility, adoption, acceptability, fidelity, potential effectiveness, coverage, their readiness for piloting, and the suitability of the training materials.

Training of health officers and nurses will include delivery of integrated wound care and psychosocial support; training of people with chronic wounds in self-care; awareness-raising for health centre attendees; detection and assessment, treatment and care initiation; referral to providers of institutional-based rehabilitation; and enhancement of institutional culture around continued learning. Training materials will include Chronic Wound Management Guidelines, the Skin-NTDs app (WHO/NLR), and simple wound management and self-care using online training materials, dermatological packages, guides and tele-dermatology [47].

For the HEWs, the training will include: delivery of wound care for people with chronic wounds, including training patients in self-care; referrals to health facilities; community awareness-raising and mobilization; active case detection; continuing care; community-based rehabilitation; and monitoring and evaluation.

Training materials at the community level will include pictorial pocket booklets, posters based on type of skin lesions/wounds, pictorial pocket booklets and posters promoting self-care. Tasks for HEWs will include screening community members for suspected skin-NTDs and other diseases with chronic wounds, community education, and advice on self-care and prevention of recurrence for people affected by chronic wounds [47].

The training materials will be evaluated and revised through change in KAP questionnaires, and an evaluation of the delivery of the training.

The topic guides and interview questions for the key informant interviews and focus groups, the questionnaires for evaluation of the training and the materials for the training will be developed once Stage 1 has been completed based on the Stage 1 activities.

The draft care package will be revised further based on the results of the pre-piloting before being piloted in one sub-district.

#### Pilot in sub-district

All wound care package interventions will be piloted at all three levels of the health system in one sub-district. As a first step, this will involve training of health professionals and other stakeholders within the district at each level of the health system, including:

- ‘Training of Trainers’ (ToT) for staff at the health care organization level, to enable them to deliver training to zonal and district level health office staff in wound care for people affected by chronic wounds due to NTDs and other conditions. The ToT will be delivered by Master trainers, of which we will have an adequate number. These Master trainers will cascade the training, which will help with the sustainability of the care package even after the project ends. The Ministry of Health (MoH) has a range of “Continuing Professional Development” (CPD) learning activities through which health professionals maintain and develop throughout their career, to ensure that they retain their capacity to practice safely, effectively and legally within their scope of practice. Our research project will embed the Master Trainer Manual into the MoH CPD training manual. We will submit our aligned manual to the MoH so that the MoH officially endorses and approves the training manual (as the MoH has clear guidelines as to how the training manual should be formatted). Once the MoH endorses our aligned manual, it will incorporate the Chronic Wound Manual in its CPD programme, which will ensure that the training will continue beyond the project’s life. This will mark a significant milestone in strengthening the quality and uniformity of chronic wound care services nationwide.
- ToT for staff at the health care organization level, to enable them to deliver training to zonal and district level health office staff to supervise facility-based health officers, nurses and HEWs. The ToT will be delivered by a Master trainer.
- Zonal and district level health office staff, to enable them to train and supervise facility-based health officers, nurses and HEWs. This training will be facilitated by staff at the health care organization level (i.e. recipients of the above ToTs), and will involve training in supervision, as well as wound care for people with NTDs and other conditions.
- Health centre staff (i.e. health officers and nurses), trained by zonal and district level health office staff.
- A multi-disciplinary team comprising senior health care workers on chronic wound management, and senior pharmacists, to enable them to provide clinical mentoring for facility-based health officers and nurses. This training will involve training in mentoring, as well as wound care for people with chronic wounds due to NTDs and other conditions.
- Pharmacy staff who manage and receive wound care supplies in health facilities. The training will cover supply chain management and will be facilitated by a pharmacist.

All training elements (in comprehensive chronic wound care, supportive supervision and supply chain management) will be evaluated through pre-post change in KAP questionnaires, and an evaluation of the delivery of the training. The questionnaires for evaluation will be the same ones used during pre-piloting, though these may be refined and improved based on the pre-piloting findings. Based on the evaluations, the training materials may be improved further ahead of the scale-up in Stage 3.

Following training, all interventions included in the wound care package (as outlined in the ‘Care package description’ section above, refined further based on the Stage 1 activities) will be implemented, to assess their adoption, feasibility, potential effectiveness, fidelity, and coverage. This will be assessed qualitatively through observation, through key informant interviews and focus groups with stakeholders who received the training and delivered the interventions, and with recipients of the interventions. We will also collect before-and-after (pre-post) quantitative data on the number of cases identified and treated by health facility staff and HEWs, and patient- and community-level outcomes through surveys. To provide a preliminary estimate for contact coverage in the pilot district, the number of cases identified and treated will be divided by the total population in need of services (this latter data will be taken from previous prevalence studies). Before we start stage three, we will evaluate the success criteria based on the assessment criteria for stage two as indicated in Table 3 below.

**Table 3:** Stage 2 success criteria and measures of assessment

Success criteria	Assessment of criteria
Wound care package successfully trialled and piloted	Piloting of wound care package completed in one health facility in Gurage zone, including the following activities: • Training of health staff and health extension workers using a stepped training ‘Training of Trainers’ (‘ToT’) approach • Implementation of care package interventions at the health care organization, health care facility and community level • Mixed-methods evaluation through: assessing number of cases identified and treated; patient cohort and community surveys (pre-post); focus group discussions; interviews; observations; and evaluation of the training • Workshop with FMOH to finalise the wound care package based on the piloting
Monitoring and evaluation plan developed for the subsequent scale-up of implementation of the wound care package	Monitoring and evaluation plan finalised and agreed by the project team for the subsequent scale-up of the wound care package, based on the activities listed above
Critical contextual factors (including drivers and barriers) identified that influence the process of integration of the wound care package into government-run health services	Qualitative assessments (i.e. focus group discussions and interviews) completed, identifying the critical contextual factors influencing integration of the wound care package, key features of the interventions influencing integration, and key health system stakeholders (as well as their coordination and capacity-building needs).
Key features identified of the interventions that influence the manner of integration into the health care system	
Key health system actors identified that have a stake in the integration of the care package into the government-run health services, and coordination and capacity-building needs identified	
Care package considered adoptable, feasible, (potentially) effective and of high fidelity by key stakeholders when integrated into government-run health services	• Adoption: Assessed qualitatively through focus group discussions and interviews • Feasibility: o Of implementing the care package (including Health Extension Worker’s capacity): Assessed qualitatively through focus group discussions, interviews and observations o Feasibility of collecting data, including cost/economic data: ▪ Data sets complete, with no substantial missing data: if < 5% missing, go ahead; if 5–10% missing, make minor procedural changes; if > 10% missing, rethink data collection methods ▪Target sample size recruited (patient cohort: *n* = 126; community survey: *n* = 200): if > 90% recruited, go ahead; if 80–90% recruited, make minor procedural changes; if < 80% recruited, rethink data collection and/or sample (e.g. inclusion criteria) ▪Sample retained: if < 5% drop-out between baseline and 3-month follow-up, go ahead; if 5–15% drop-out, make minor procedural changes; if > 15% drop-out, rethink data collection methods • Potential effectiveness: o Improvements observed between baseline and 3-month follow-up for at least two of the following outcomes: wound size, pain associated with wound, disability (WHODAS), quality of life (WHOQOL), depression (PHQ-9), anxiety (GAD-7), experienced discrimination (DISC), social distance by the community (SDS) o No important worsening observed for more than one of these outcomes • Fidelity: o Assessed qualitatively through focus group discussions, interviews and observations o Training attendance: at least 80% attendance
Observable trends in the utilisation and coverage of the care package identified	• Number of cases identified and assessed: Increase observed in number of cases identified and assessed at 3-month follow-up compared to baseline • Proportion of cases identified who are then treated: If < 40% of cases treated, rethink this part of care package; if 40–60% of cases treated, make minor procedural changes; if > 60% treated, go ahead

### Stage 3

As indicated in Table 3 above, after evaluation the success criteria for stage two and assessing the implementation scalability and sustainability, stage 3 will be conducted. This stage corresponds to Phases 3 and 4 of the MRC framework on complex interventions, i.e. the evaluation and implementation. We will scale up the wound care package and its evaluation in two selected districts in Ethiopia. Scale-up will be informed by the results of Stages 1 and 2 of the study.

#### Economic analysis

The health economics analysis aims to (i) Estimate resources and costs associated with development, piloting and integrating the new wound care package into the routine primary health care system, and (ii) Assess the cost-effectiveness of this care package using a range of clinical and quality-of-life outcomes.

In Stage 1 (the development of the wound care package), we will collect data on resources involved in the development of the wound care package, i.e. situational analysis, **‘**ToC’ workshops, key informant interviews, focus groups and preparation of training materials. The costs will cover staff time spent on developing guidelines and training materials, and expenses associated with conducting interviews and workshops (e.g. venue hire, travel expenses, accommodation, food, digital equipment and stationary materials). Data will be collected using purpose-designed questionnaires and interviews with programme managers.

In Stage 2 (piloting of the wound care package), data will be collected on implementing the care package in one sub-district in Ethiopia. This will include training of health officers and nurses in delivery of integrated wound care and psychosocial support, detection and assessment, treatment and care initiation, referral to providers of institutional-based rehabilitation, training of people with chronic wounds in self-care, awareness-raising for health centre attendees and enhancement of institutional culture around continued learning. We will collect data on the costs of training (e.g. trainers and trainees time, training materials, digital equipment, travel and accommodation); leaflet and poster preparation and distribution; wound treatment supplies (e.g. soap, ointment, bandages, gloves, gauzes, antiseptics and antibiotics); storage and delivery of treatment supplies, and telehealth gadgets. Data will be collected from financial documentation, purpose-designed questionnaires and interviews with programme managers.

In Stage 3 (scale-up of the wound care package in two selected districts in Ethiopia), we will collect data to inform the cost-effectiveness analysis of the implementation of the wound care package. The data will include resources and costs related to the delivery of the interventions and the use of healthcare services used by people with chronic wounds. The services will include contacts with healthcare specialists and traditional healers, hospitalisations, medication, traditional remedies, and out-of-pocket expenses related to the management of chronic wounds. We will collect data on the number of days when individuals were completely unable to work because of their wounds (or go to school) and the number of days when they experienced some difficulties when working (or attending school). We will also collect data on money borrowed by families over the past year to cover costs of treatments and living. A cost-effectiveness analysis will be performed using a range of clinical and quality-of-life outcomes collected during the implementation research study to inform decision-makers whether the new care package provides good value for money.

#### Data management and analysis

All data analyses will be conducted by members of the EnDPoINT II research team at BSMS and CDT-Africa, AAU.

During Stage 1, the documents identified through the scoping review will be summarised systematically into a narrative review and will be used to guide the development of the wound care package. Data from the situational analysis and resource assessment will be described quantitatively as frequencies or qualitatively (free-text data). A ‘ToC’ map and an accompanying narrative will be produced based on the ‘Theory of Change’ workshops.

Key informant interviews and focus group discussions during Stages 1 and 2 will be audio-taped, transcribed in Amharic and then translated into English with independent translation checks of selected interviews. The data will be analysed using thematic analysis, either manually or with the assistance of a qualitative software package (NVivo). Validity of data will be assessed through the identification of initial codes by an external experienced qualitative researcher, agreement over the framework through which the data will be presented, presentation of initial findings to experienced academics to assess plausibility, and single counting of identified events. Validity assurance will also involve checking of alignment of research questions, interview guides and sampling procedures. The observation data collected during Stage 2 will be recorded on paper and will be analysed in a similar way.

For quantitative data collection and storage during Stages 2 and 3, we will use REDCap, a secure web application for building and managing online surveys and databases. Data quality control processes will be agreed and implemented. Participant progress through the pre-post study will be shown using a flow diagram according to the CONSORT Statement 2010 extension for pilot and feasibility studies [48] adapted to reflect the non-randomised nature of our study. Data will be summarised descriptively using means and standard deviations for normally distributed variables, medians and interquartile ranges for skewed continuous variables and frequencies and percentages in each category for categorical variables. Amount of missing data will be summarised; no imputation will be conducted. Change from baseline in patient-centred outcomes and other point estimates will be presented with 95% confidence intervals.A full statistical analysis plan will be agreed prior to database lock and final analysis.

Wound care clinics will be set up in health facilities in two selected districts in Ethiopia. Setting up this new service will involve the review of all wound assessment tools, patient documentation, referral forms and patient management systems and audit processes. A plan for evaluating the service will be developed using data (e.g. healing rates, wound size improvement, origin of referrals, number of infections and client satisfaction). The results from each of the activities in Stages 1 and 2 will be triangulated to develop and finalise the wound care package and strategies for its integration into the routine health care delivery system in selected districts/zones in Ethiopia.

## Discussion

This implementation research is aimed at designing and testing an integrated chronic wound care due to skin-NTDs and other chronic health conditions in the primary health care system of Ethiopia. In the meantime, it will explore challenges and enabling conditions for scale-up of chronic wound care within routine primary health care services in Ethiopia that will substantially improve access to essential care and support for affected persons. The overall implementation of an integrated chronic wound care is guide by the national [49]; and the WHO’s skin-NTDs management protocol [50]. Additionally, the integration of chronic wound care due to skin-NTDs and others NCD conditions in the primary healthcare system of Ethiopia has immense contribution in optimizing the community-based screening of NTDs using HEWs and managing cases through primary health care unit healthcare system [51]. The capacity-building interventions to strengthen primary healthcare-based integrated detection, management and reporting of chronic skin-NTDs is also designed through core principles of the instructional design training manual of the Ethiopian MoH which will help the scalability and suitability of the approach.

The evidence generated through this research will likely contribute to policy and practice. The study will use an embedded implementation research approach that comprises a formative study and evaluation of the holistic integrated chronic wound care due to skin-NTDs and other neglected chronic health conditions. Substantial context-driven evidence will be generated from this implementation research that will help to inform policy and programs in Ethiopia and similar contexts in Africa. Knowledge products from this study will be disseminated using a variety of channels, including publications in scientific journals, conference presentations, policy briefs, and workshops. All relevant stakeholders in the primary health care setting including persons affected by chronic wounds, healthcare providers, program planners, and policymakers will benefit from this study.

## Data Availability

Data sharing is not applicable to this article as no datasets have been generated or analysed.

## Abbreviations

AAU: Addis Ababa University
BSMS: Brighton and Sussex Medical School
CDT-Africa: Center for Innovative Drug Development and Therapeutic Trials for Africa
CSA: Central Statistical Agency
EnDPoINT II: Excellence in Disability Prevention Integrated across NTDs – Part II
HAD: Health Development Army
HEWs: Health Extension Workers
HICs: High-Income Countries
KAP: Knowledge, Attitudes and Practices
LF: Lymphatic Filariasis
LMICs: Low- and Middle-Income Countries
MHealth: Mobile Health
MoH: Ministry of Health
MRC: Medical Research Council
NCDs: Non-Communicable Diseases
NGO: Non-Governmental Organization
NTDs: Neglected Tropical Diseases
NIHR: National Institute for Health and Care Research
PAG: Public Advisory Group
REDCap: Research Electronic Data Capture
ToC: Theory of Change
ToT: Training of Trainers
UK: United Kingdom
WHO: World Health Organization
